# Is There a Link between Suckling and Manipulation Behavior during Rearing in Pigs?

**DOI:** 10.3390/ani11041175

**Published:** 2021-04-20

**Authors:** Friederike K. Warns, Mehmet Gültas, Astrid L. van Asten, Tobias Scholz, Martina Gerken

**Affiliations:** 1 Department of Animal Science, Ecology of Livestock Production, University of Goettingen, Albrecht-Thaer-Weg 3, 37075 Goettingen, Germany; mgerken@gwdg.de; 2 Department of Animal Science, Breeding Informatics Group, University of Goettingen, Margarethe-von-Wrangell-Weg 7, 37075 Goettingen, Germany; 3 Center for Integrated Breeding Research (CiBreed), Albrecht-Thaer-Weg 3, Georg-August University, 37075 Göttingen, Germany; 4 Faculty of Agriculture, South Westphalia University of Applied Sciences, Lübecker Ring 2, 59494 Soest, Germany; 5 Department of Animal Production, Agricultural Chamber of North Rhine-Westphalia, Haus Duesse, 59505 Bad Sassendorf, Germany; astrid.vanasten@lwk.nrw.de; 6 Agricultural Test Center VBZL Haus Duesse, Agricultural Chamber of North Rhine-Westphalia, Haus Duesse, 59505 Bad Sassendorf, Germany; tobias.scholz@lwk.nrw.de

**Keywords:** pigs, tail biting, suckling behavior, dominance, rearing period, manipulation behavior

## Abstract

**Simple Summary:**

Tail biting, a well-known problem in modern pig production, reduces pigs’ welfare and causes economic losses. It is influenced by several external and internal factors, such as housing condition, management, genetics, and age of the animals. Within the internal factors, the individual predisposition to tail biting is difficult to identify. In our study, we analyzed the manipulation behaviors of weaner pigs and their relationship with agonistic behaviors of the piglets during suckling to identify groups of piglets which showed similar suckling and rearing behaviors. In our experiment tail biting increased at the middle and end of rearing. Most animals were observed as both biters and victims of tail biting. During our observations, we found indications that tail-biting pigs showed mainly submissive behavior in teat disputes. These pigs might compensate their submissiveness by biting tails to chase other pigs from resources with restricted access, such as feed or enrichment material. Further research should consider more aspects of a pigs’ personality suitable for early identification of pigs predisposed for later tail biting. This early identification would allow intervention measures to be taken earlier, thereby reducing tail biting and its consequences.

**Abstract:**

Inadequate possibilities to perform oral manipulation behavior for pigs can lead to misdirection and thus tail biting. Our study aimed to analyze manipulation behaviors of weaner pigs with focus on tail biting and the relationship with agonistic characteristics of the piglets during suckling. We analyzed the individual manipulation behavior of 188 weaner pigs. General health condition and tail lesions were determined weekly. Correlations were estimated between weight at weaning and at the end of rearing period, frequency of manipulative rearing behaviors and Dominance and social tension index based on suckling behavior. Principal component and cluster analyses were performed to identify groups of piglets which showed similar suckling and rearing behaviors. Tail biting increased at the middle and end of rearing with switching roles of biters and victims. Tail lesions were correlated with received tail biting behavior but occurred with a delay of more than a week. The frequency of performed tail biting was correlated with dominance index (r_s_ = −0.256, *p* < 0.01) and weaning weight (r_s_ = −0.199, *p* < 0.05). We assume that performed tail biting is more often observed in pigs who show mainly submissive behavior in teat disputes.

## 1. Introduction

Pigs spend a significant amount of their time using oral manipulation to explore objects around them by sniffing, digging or chewing. This behavior can be redirected to other conspecifics, especially in barren housing systems [1,2], and can lead to various behaviors such as tail biting, ear biting or belly nosing [3]. Tail biting is one of the most important problems in modern pig production. Beside economic losses due to carcass trimming or full loss as consequence of spread infection, it causes stress, painful injuries and thereby reduced animal welfare in affected pigs [4,5,6]. 

Three different forms of tail biting are distinguished by the behavior of the performing pig: “two-stage tail biting”, “sudden-forceful tail biting” and “obsessive tail biting” [7]. A tail biting outbreak may occur with usually one pig starting to bite and thereby encouraging further pen mates to join the behavior [8,9]. Within an outbreak, switching roles between performers and victims were observed [10,11]. Analysis of tail biting behavior is difficult because it occurs relatively rarely and seems unpredictable [12]. A wide range of influencing factors have been discussed by several authors (e.g., [4,7,9,13,14,15]). For example, refs. [10,16,17] found lighter piglets to be more likely to bite pen mates and heavier piglets were bitten more frequently, whereas [18] found tail biters to be heavier than non-tail-biters. However, several authors failed to confirm significant correlations between a pigs’ body weight and its frequency of performed tail biting [11,19,20]. 

Several solutions were evaluated to prevent a tail biting outbreak such as environmental enrichment. Fewer wounded tails, more exploration behavior and improved animal welfare were found when pigs were offered organic objects and substrate which is ingestible, smellable, chewable, deformable, and destructible like roughage, wood shavings, or peas as enrichment material (e.g., [5,21,22,23]). 

Another approach is to establish methods for early detection of occurrence of tail biting such as scoring of tail lesions or changes in behavioral traits. Tail biting outbreaks often seem to begin with first visible tail lesions two to three weeks after weaning, with the development of the percentage of injured pigs and the severity of the injuries depending on the design of the barn, including the enrichment material offered [9,24,25,26,27,28,29]. In many cases, these lesions are recognized with a delay of a few days after the frequency of tail biting behavior has already increased [30].

When analyzing the nosing behavior of pigs, Camerlink and Turner [31] found a low percentage of social nosing behavior that were followed by subsequent potentially injurious oro-nasal behavior. Within these behaviors, tail biting, ear biting and belly nosing were correlated with nosing of the corresponding body parts. In further studies positive correlations between tail biting and other manipulation behavior were found with biters spending more time to manipulate enrichment material than victims [10,32] and also tended to bite more parts of the body [32,33] than non-tail-biters. In contrast, Larsen et al. [34] found no alteration of object manipulation prior to an outbreak but overall lower object manipulation rates in pens with tail damage. Furthermore, increased tail-in-mouth behavior [30,35] and activity level [10,33,34], as well as an alteration of tail posture prior to a tail biting outbreak [36,37,38,39,40,41], are discussed as signs for early detection of tail biting.

Little is known whether predispositions for later tail biting can be already deduced from the piglets’ behavior during the suckling period. Prunier et al. [12] reviewed several predisposing factors for biting experienced in early life as for example social stress due to fighting for teats or other resources. The authors pointed out an effect of this stress on aggressive biting during hierarchy formation but could not transfer this effect to non-aggressive forms such as tail biting [12]. Furthermore, Hoy et al. [11] did not find an association of certain teat positions during suckling with performed tail biting during rearing.

In the present study we followed individual agonistic behavior from suckling until the end of the rearing period at the age of 70 days. Analyses of suckling behavior is described in detail in Warns [42]. Here, we report on manipulation behavior of the weaned pigs with a special focus on performers and victims of tail biting across the rearing period of six weeks. Evaluation of general health condition and possible tail lesions of the pigs further provided additional information to the behavioral analysis. In a further step a possible relationship between agonistic behavior shown by a piglet during the suckling bouts and its manipulation behavior during rearing was analyzed. From these results, we wanted to deduce whether agonistic behavior shown during the competitive suckling situation could be used to predict tail biting during the later rearing phase.

## 2. Materials and Methods

The study was carried out at the Agricultural Test Center VBZL Haus Duesse of the Agricultural Chamber of North Rhine-Westphalia in Bad Sassendorf, Germany, between August 2017 and March 2018. The animals were kept according to the German Animal Protection Law and the Animal Welfare Livestock Farming Regulation.

The experiment was carried out in two repetition groups with a time interval of five weeks between trials. During suckling, twelve litters were housed in conventional farrowing crates (4.58 m^2^ per crate) and included 174 piglets across both repetitions. The environmental temperature during suckling was automatically regulated by forced ventilation and set at 23 °C. The piglets had free access to a piglet nest with a heated floor plate, which was completed by a heat lamp for the first 48 h after birth. Usually, the animals had artificial light between 07:00 h and 16:00 h in addition to daylight via windows. During the entire suckling period the piglets had free access to water by a drinking trough. No enrichment material was offered to the piglets during suckling. Agonistic suckling behaviors of the piglets were analyzed individually (further details see [42]). Out of these piglets, we chose 59 weaner pigs per repetition for analysis of rearing behavior with a total of 118 pigs (59 castrated males and 59 females; Table 1) across both repetitions. The non-tail docked pigs were crossbreds of a Piétrain sire and a Topigs20 or Topigs70 dam and had an average birth weight of 1.44 ± 0.34 kg. Pigs were weaned with an age of 28 days and an average weight of 7.80 ± 1.44 kg. From day 21 after birth the pigs had ad libitum access to a feeding trough with a commercial rearing feed (feed A; 13.6 MJ ME, 15.0% CP, 1.40% Lys, 0.20% Na).

From all pigs who reached a weaning weight of 6 kg or more, 59 pigs per repetition were chosen and distributed to rearing pens with nine (one pen) to ten pigs (five pens) each. By composing the groups, we considered similar weaning weight, a maximum of three pigs from the same litter and a balanced sex ratio in each group (Table 1).

The conventional rearing pens for ten pigs were sized 2.1 × 1.7 m (0.36 m^2^ per pig) with a fully slatted floor and no bedding material. In two pens, the available space was smaller because of a ventilation shaft, so only nine pigs were housed in these pens (0.37 m^2^ per pig). The environmental temperature was automatically regulated by forced ventilation and set on 29 °C at the beginning of rearing (day 29 of life). During the subsequent weeks, it was decreased stepwise until 24.5 °C at the end of rearing (day 70 of life). Usually, the animals had full artificial light (intensity: 90 lux in average) between 07:00 h and 16:00 h additionally to daylight. At the end of the rearing period, the pigs had an average weight of 22.80 ± 3.10 kg.

During the rearing period, the animals had ad libitum access to feed in a feeding trough and fresh water by drinking nipples. Feeding took place automatically by a dry feeding system. Until day 49 of life, the pigs received rearing feed A which they were used to from the last week of the suckling period. From day 50 of life, the diet was gradually changed during the next four days to rearing feed B (13.4 MJ ME, 16.0% CP, 1.31% Lys, 0.20% Na) which was fed until the end of the rearing period. The animal to feeding place ratio was 3:1 in pens with nine pigs (3.31 m^2^) and 3.3:1 in pens with ten pigs (3.56 m^2^). In each pen there was a metal chain with a plastic piece and a cotton rope provided as enrichment material. Twice a day, about 200 g of pellet mix of alfalfa, hay, corncob, and straw pellets were added to a pig bowl in each pen for oral manipulation. If tail biting occurred, pigs were offered a jute sack attached to the wall of the affected pen as additional enrichment material.

The individual body weight was measured weekly outside of the pens on a commercial stationary digital pig weight scale to the nearest 100 g (piglet scale, Meier-Brakenberg).

Possible tail lesions of each individual pig were scored weekly by the same observer during weighing. The individual general health condition was scored weekly in the pen using a scoring scheme modified from the Welfare Quality Assessment Protocol^®^ for Pigs (Ref. [43], Table 2) and included lameness, injury of the ears, the carpal joints, and the body sides.

The scoring scheme for possible tail lesions (modified from FLI [44]; Table 3) included tail length, hair coat, cleanliness, skin perforation, blood, and necrosis.

During the entire suckling period each pen was equipped with a stationary HD camera (Dallmeier DF4820HD-DN/IR, 720 p, 6 fps) and the pigs were filmed continuously over the entire suckling phase of four weeks. The behavior of the pigs during 30 suckling bouts was analyzed for each pig individually as described in Warns [42]. In brief, based on the occurrence of agonistic traits during suckling, a dominance index [45] and social tension index [46] was calculated for each piglet individually. The dominance index was calculated by dividing the difference between the number of winds and defeats of social interactions by the sum of wins and defeats of social interactions [45]. For the social tension index, the difference between the sum of all the aggressive actions an animal has performed and the sum of all the aggressive actions an animal has received was calculated [46]. A positive dominance index of a piglet was the result of a high rate of success in teat disputes, whereas a high positive social tension index expressed a high willingness to initiate a teat dispute to gain access to a teat.

Similarly, during rearing, each rearing pen was equipped with a stationary HD camera (Dallmeier DF4820HD-DN/IR, 720 p, 6 fps) and the pigs were filmed continuously over the entire rearing phase of six weeks. For evaluation of the individual behavior of the pigs, each animal was marked with individual geometric symbols on its back using a commercial animal marking spray (Raidex^®^, Raidex GmbH, Dettingen, Germany; red, green and blue). The first marking took place directly after transfer to the pens. The pigs were remarked every two to three days to avoid fading of the marks. To ensure individual identification of pigs, only videos taken during the time with artificial light (07:15 to 16:00 h) were chosen for further analysis. In each repetition, we analyzed two days per week resulting in a total of twelve observation days per pig and repetition (total of 24 observation days across both repetitions and pens).

Videos were analyzed by the same observer for each pig individually by using the software Mangold Interact^®^ (Version 14.3.9.0; Mangold International GmbH; Arnstorf, Germany). Six different behavioral patterns were coded, separated in animal directed and object directed behavior with three behavioral patterns each. The animal directed behavior included tail biting, ear biting and belly nosing. Tail and ear biting were recorded continuously within the first ten minutes of an hour from 08:00 h to 15:10 h, including information of the performer and the victim. Additionally, the reaction of the victim of tail biting was noted. For belly nosing and the three object directed behavioral patterns (manipulation of the rope, the pellets in the pig bowl, and the jute sack) the frequency of occurrence was determined by instantaneous scan sampling with a 30 sec interval within the above-mentioned time frame. For these four behaviors, the performer was noted (Table 4).

The statistical procedures were performed with R (Version 1.2.1335, RStudio, PBC, Boston, MA, USA). For analyzing general health condition and tail lesions of the rearing period we converted the evaluated scores to binary data and compared the different scoring times by chi-square tests (function “chisq.test”; [47]). For illustration of the development of behavioral traits during the entire rearing period, manipulation behaviors were analyzed by calculating average frequencies per pig, hour, and observation day. For the continuously recorded tail and ear biting, we determined the individual frequency of the behavior within the first ten minutes of an hour, extrapolated this result to the total hour by multiplying the result by six (according to Zonderland et al. [30]) and then calculated the average frequency of behavior per pig, hour and observation day. The remaining manipulation behaviors (recorded by scan sampling) were calculated as percentage of sampling points per hour and day in which behaviors were shown. For this purpose, we divided the determined frequency of behaviors shown at sampling points by the amount of sampling points per observation day and calculated an average per pig, hour and observation day.

Further statistics were based on the frequencies of behavior per individual, which were summarized per behavior over the entire observation period. Analyses of variance were performed for performer and victim of tail and ear biting, belly nosing, rope, pellets and sack manipulation (function “aov”; [48]) with repetition, sow, sex and pen of the pigs as fixed effects. Parameter “sow” referred to the sow pigs were raised at, so it described not necessarily the genetic mother of the pig. Tukey post-hoc tests were performed by function “TukeyHSD” [49]. Additionally, Spearman rank correlation coefficients were calculated (function “cor.test”; [50]) with a confidence interval of 95% between weaning weight, weight at the end of rearing period, frequency of analyzed manipulation behaviors during rearing and dominance index and social tension index of the suckling piglets. Scatterplots were used to visualize the relationships between dominance index, social tension index and performer and victim of tail biting by the function “ggplot” (package “ggplot2”; [51]). Principal component analysis was performed to evaluate relationships between the behavior traits of the suckling and rearing period (function “PCA” of package “FactoMineR”; [52]) and its result was visualized in a scree plot for percentages of explained variances (function “fviz_eig” of package “factoextra”; [53]) and bar plots (function “fviz_contrib” of package “factoextra”; [53]) for contribution of variables and individuals to Dimension 1–2. Cluster analysis was performed with function “eclust” (package “factoextra”; [53]) to determine two clusters. Results of cluster analysis were visualized with function “fviz_silhouette” (package “factoextra”; [53]). A high positive silhouette value (S_i_) represented the similarity of an individual with its own cluster and poor matching with other clusters. Finally, principal component analysis was performed within the two calculated clusters as described above. Level of significance was set at *p* < 0.05. Values are means ± SD if not otherwise stated.

## 3. Results

### 3.1. Weight Gain and Results of Injury Evaluation

Pigs were assigned to the pens according to their weaning weight. Average daily weight gain was similar in both repetitions with 0.362 ± 0.064 and 0.355 ± 0.066 kg in repetitions 1 and 2, respectively.

Evaluation results of general health condition and tail lesions were analyzed as binary system to describe the presence or absence of an injury (Table 5). The proportion of pigs with injured body sides increased in both repetitions (0 to 71% and 37 to 59%, respectively) whereas the proportion of pigs with injured carpal joints decreased until the end of rearing period in both repetitions (61% to 5% and 49% to 10%, respectively). In both repetitions a high variation within the proportion of pigs with injured ears (2% to 22% in repetition 1 and 3% to 80% in repetition 2) occurred whereas the frequency of lameness was very low.

Overall, the proportion of pigs with tail lesions increased in both repetitions until the end of the rearing period with considerable variations between repetitions. The proportion of pigs with tails with fresh blood ranged between 15–36% and partial losses were noted in 15–29% of the animals at the end of the rearing period.

### 3.2. Development of Manipulative Behaviour during the Rearing Period

Analyses of variance showed a significant effect (*p* < 0.001) of repetition on frequency of received tail biting with a higher average in repetition 1 (0.35 ± 0.25 received tail biting behaviors per hour and pig) than in repetition 2 (0.20 ± 0.14 received tail biting behaviors per hour and pig). In addition, a significant effect (*p* = 0.008) of pen with a high average in pen 5 of repetition 1 (0.61 ± 0.37 received tail biting behaviors per hour and pig) was found. The frequency of performed tail biting was not affected by repetition, sow, sex, and pen (Table 6).

**Figure 1:**
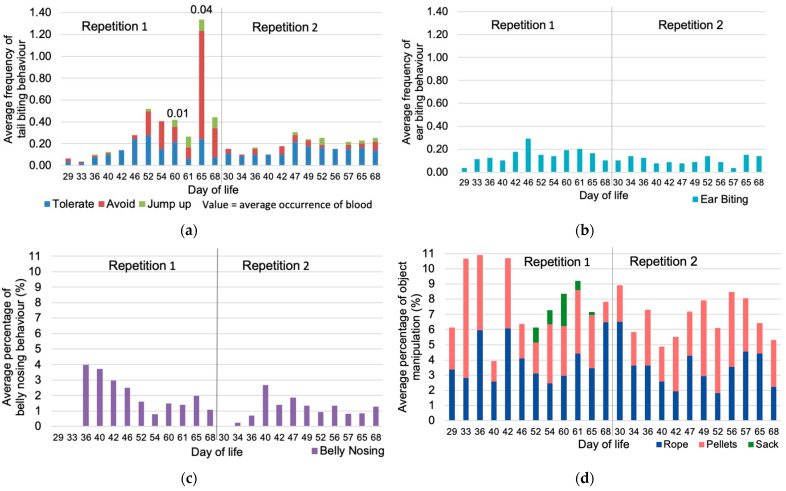
Average frequency and percentage of manipulation behavior per pig and hour by observation day of the rearing period by repetition (**a**) average frequency of tail biting with victims’ reaction per pig; (**b**) average frequency of ear biting per pig; (**c**) belly nosing behavior per pig as percentage of sampling points; (**d**) object manipulation per pig as percentage of sampling points.

In the first repetition the average frequency of tail biting incidences per pig and observation day increased stepwise until a first peak at day 52 of life (0.52 tail biting behaviors per hour and pig) and reached its maximum at day 65 of life (1.37 tail biting behaviors per hour and pig; Figure 1a). Until the first peak the majority of tail biting incidents were tolerated by the victim. Subsequently, the proportion of responses “avoid” or “jump up” by the victim increased until the end of the rearing period. Tail biting behavior resulting in fresh bloody injuries was observed only rarely. In repetition 2, the average frequency of tail biting behavior varied between observation days with a maximum of 0.31 tail biting incidents per hour and pig on day 47 of life and mostly tolerating reactions of the victims.

The average frequency of ear biting varied on a low level between observation days (Figure 1b). The average frequency of ear biting was significantly higher in repetition 1 (0.15 ± 0.12 behaviors per hour and pig) than in repetition 2 (0.10 ± 0.10 behaviors per hour and pig) but not influenced by further tested effects.

Among belly nosing behavior (Figure 1c) the highest average frequencies per sampling points was observed in both repetitions in the second week after weaning (days 36 and 40 of life), followed by varying but overall decreasing proportions until the end of the rearing period. Belly nosing was affected by repetition (*p* = 0.005) with a higher average in repetition 1 (0.36 ± 0.37% of sampling points) than in repetition 2 (0.23 ± 0.22% of sampling points), and was also affected by pen with a significantly (*p* = 0.002) high average in pen 2 of repetition 1 (0.62 ± 0.45% of sampling points), and by sow.

All object directed manipulation behaviors (“rope”, “pellets” and “sack”; Figure 1d) were observed in varying frequencies and affected by sow. Rope manipulation behavior was also significantly affected by sex and pen with higher averages shown in male (4.09 ± 2.04% of sampling points) than in female pigs (3.40 ± 1.78% of sampling points) and a significant low average in pen 6 of repetition 1 (2.46 ± 0.57% of sampling points). Manipulation of the sack was only observed on days 52 to 65 of life in three pens of repetition 1. The sack was only offered in three pens of repetition 1 with severe tail biting, which strongly biased the effects of repetition, sow and pen.

### 3.3. Dominance and Social Tension Index from the Suckling Period

For allocation to the rearing pens at weaning, littermates were distributed across different pens. The resulting mean indices per rearing pen (Table 7) showed high standard deviations. Highest mean dominance indices were found in pens 5 and 6 of repetition 1 whereas the mean social tension index was lowest in pen 2 of repetition 1 (Table 7).

### 3.4. Correlations between Traits

**Figure 2:**
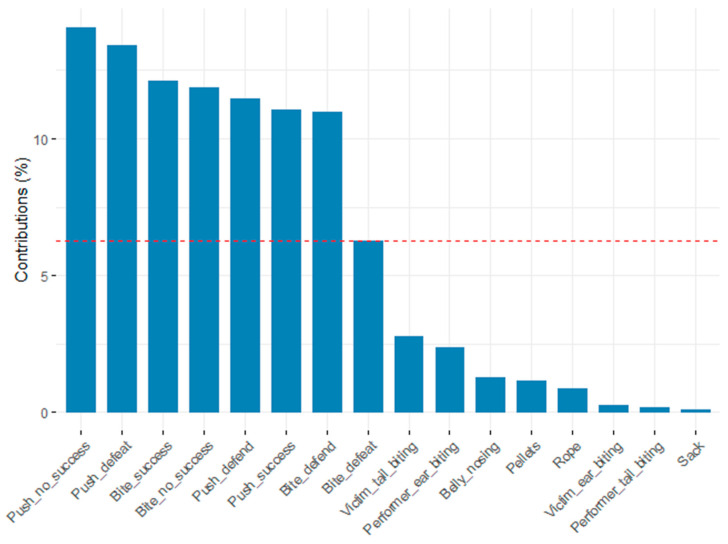
Contribution of variables to principal component factors 1 and 2 for all pigs; dashed line = expected value if contribution were uniform.

Most correlations were of low magnitude (Table 8). A high correlation was found between body weights at the age of four and ten weeks. Among traits recorded during suckling, dominance index was closely negatively correlated with social tension index (r_s_= −0.466, *p* < 0.001; Table 8). However, the relationship between dominance index and social tension index with the manipulative behavior during rearing was low. Only between dominance index and tail biting performer a moderate negative correlation of −0.256 (*p* = 0.005; Figure 2) was found. Traits of tail lesions were consistently correlated with tail biting receiver (r_s_ = 0.265 to 0.379). The correlation with “sack” is biased, because only in the case of tail biting occurrence was a sack provided.

### 3.5. Principal Components and Clusters

Table 9 lists the outcomes of the principal component analysis (PCA) performed on the frequency of agonistic suckling behaviors and manipulative rearing behaviors. Five main factors were identified with Eigenvectors >1, which together explained 54.953% of the variation between pigs. Principal component factors 1 and 2 explained 30.572% of the variation between the pigs (Table 9). The highest contributions to these principal components were shown by seven of the eight suckling behaviors (Figure 2).

**Figure 3:**
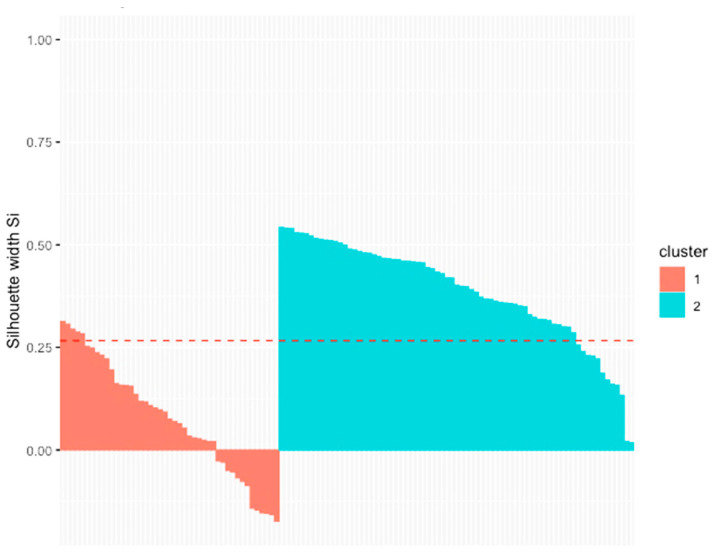
Cluster silhouette plot for categorization of piglets in two clusters based on their suckling and rearing behavior; dashed line = average S_i_.

We performed a cluster analysis to identify similar groups of pigs based on their observed behaviors during both suckling and rearing. However, the visualization of the results in a silhouette plot (Figure 3) showed an overall low similarity of the pigs with their cluster (average S_i_ = 0.27).

**Figure 4:**
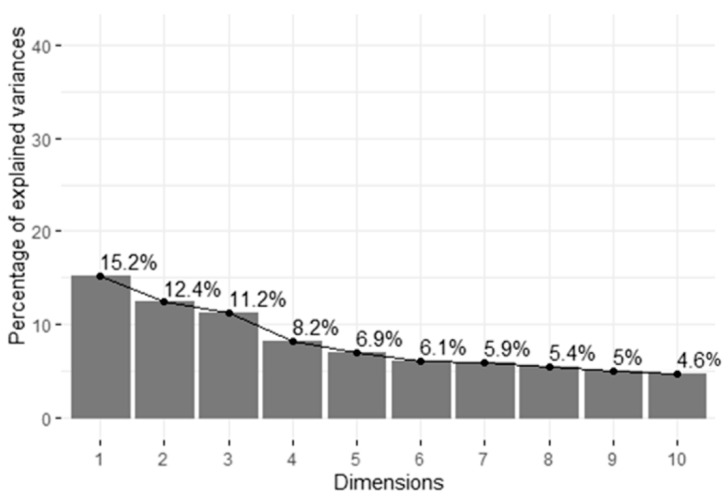
Scree plot with percentage of explained variances per principal component factors (dimensions) of cluster 1.

**Figure 5:**
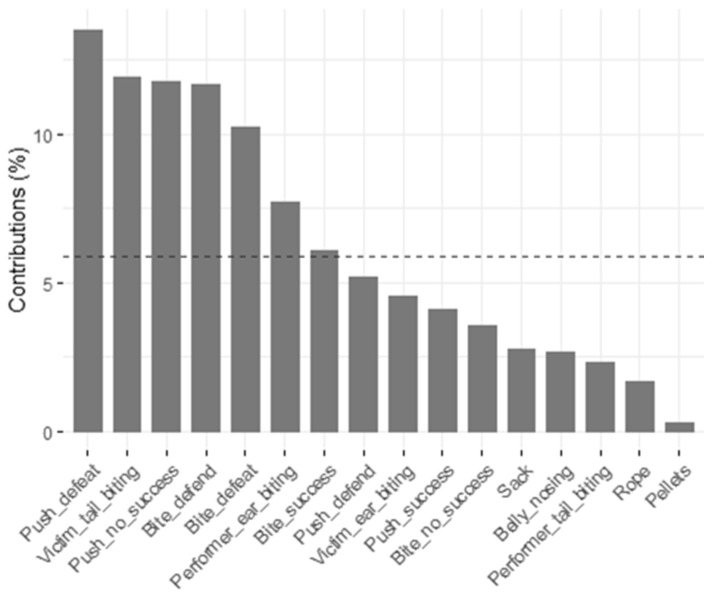
Contribution of variables to principal component factors 1 and 2 for cluster 1; dashed line = expected value if contribution were uniform.

Within cluster 1, five main factors were identified with an Eigenvalue >1, explaining 53.84% of the variation between pigs (Figure 4). The total contribution of variables to principal components 1 and 2 (Figure 5) showed highest loadings for five suckling behaviors, “victim of tail biting” and “performer of ear biting”.

**Figure 6:**
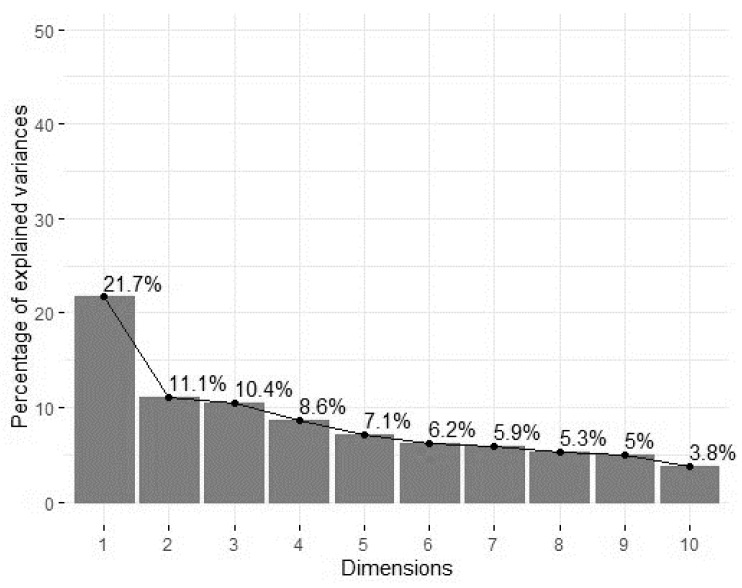
Scree plot with percentage of explained variances per principal component factors (dimensions) of cluster 2.

**Figure 7:**
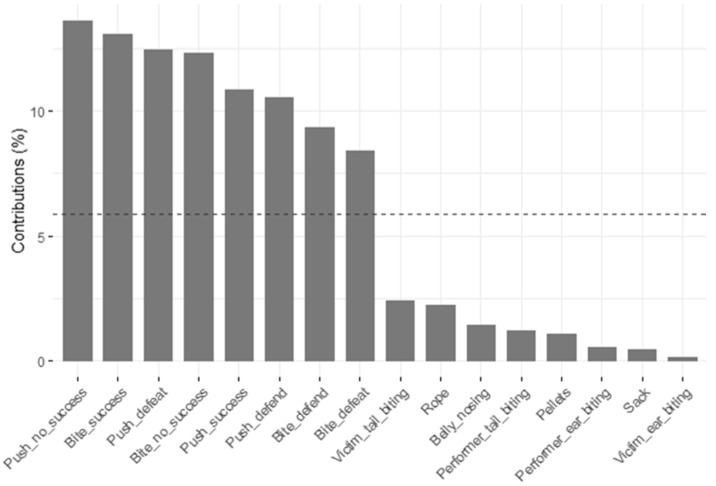
Contribution of variables to principal component factors 1 and 2 for cluster 2; dashed line = expected value if contribution were uniform.

Cluster 2 identified five main factors with an Eigenvalue >1, explaining 58.89% of the variation between pigs (Figure 6). Within principal components 1 and 2, the contribution of variables (Figure 7) showed highest loadings for the eight suckling behaviors.

## 4. Discussion

Tail biting in pigs is a complex behavior due to the high number of influencing factors, its low frequency and unpredictability. In this study, we observed manipulative behaviors in weaner pigs, including performers and victims of tail biting, and analyzed correlations with agonistic suckling behaviors. 

### 4.1. Manipulation Behaviors

The frequencies of observed ear biting and belly nosing behavior varied over the whole experiment and were also affected by repetition. As weaning entails separation of the sow, transport to a new housing environment and mixing with unfamiliar conspecifics, it goes along with new and maybe stressful impressions for the piglets [54].

Especially after regrouping of unfamiliar pigs, vigorous fights were observed between them to establish a social hierarchy. Those fights are primarily characterized by bites, and therefore lesions at the head and ears of the competitors, but can also involve the flank of the opponent [55]. In our experiment, the percentage of recorded body side and ear injuries was highest in the first week (until day 35 of life) and the last two weeks of rearing (day 63 of life until end of rearing). For the first week, we suggest that these injuries were caused by fights for hierarchical order. The percentage of injured body sides was also correlated with pigs’ weight at week ten (day 70 of life), indicating that heavier pigs were hurt more frequently. This may be explained by decreasing available space per pig with proceeding growth of the animals [56], which may explain the high percentages of body side and ear injuries in the last two weeks of rearing.

We observed highest frequencies of tail biting around day 47–52 and day 65 of life. Comparing the pens, the highest average of tail biting was reached in pen 5 of the first repetition, possibly as a result of “obsessive tail biting” performed by one specific pig [7]. This individual stood out by performing a multiple of tail biting behaviors compared to its pen mates with the maximum mean of 29.25 tail biting behaviors per hour on day 65 of life and overall, only 2.6% tolerating reaction of its victims. By defining “tail biting”, no distinction was made between tail-in-mouth and visible biting of the tail. Thus, the severity of the biting was deduced from the victim’s reaction. It is of particular interest to note that both performing and receiving tail bites was observed in most pigs as also described by Zonderland et al. and Hoy et al. [10,11]. 

No significant correlation was found between the frequency of performed tail biting and any frequency of other manipulation behaviors. The frequency of received tail bites was positively correlated with manipulation of the jute sack. This result disagrees with results of several authors who found biters to spend more time manipulating enrichment or other parts of the bodies of their pen mates than victims [10,18,32]. Nevertheless, it must be taken into account that our finding was expected, since the jute sack was only offered in pens with severe tail lesions shown by the majority of the pigs.

Within the weekly scorings, a first significant increase of the proportion of tail lesions between two consecutive evaluations was found for all tail lesion parameters on day 63 of life in repetition 1 and day 56 of life in repetition 2. In both repetitions the increase continued until the end of the rearing period. Thus, in our experiment, those lesions occurred at least a week later than described by several authors who observed a first increase of tail lesions two to three weeks after weaning (day 42 to 49 of life) [24,25,26,27,28,29]. The presence of tail lesions at the end of rearing was positively correlated with the frequency of received tail bites. However, the lesions were not diagnosed until ten days after the notable increase in the frequency of tail biting (increase of tail biting at day 52 vs. increase of tail lesions at day 63 of life in repetition 1; increase of tail biting at day 47 vs. increase of tail lesions at day 56 of life in repetition 2). This delay confirms previous findings of Zonderland et al. [30], even though it was also influenced by the interval of a week between lesion evaluations. Moreover, it underlines the importance of behavioral observations for early detection of tail biting and thereby successful prevention of severe tail lesions.

### 4.2. Relationship between Suckling and Rearing Behaviors

After transfer and regrouping to the rearing facilities, we found considerable differences between the rearing pens. We found moderate negative but significant correlations of performed tail biting behavior with dominance index and weaning weight, respectively, indicating that light subdominant pigs have performed more tail biting behavior than heavier piglets and therefore confirm findings of Zonderland et al., Beattie et al. and van de Weerd et al. [10,16,17]. The lower weaning weight might be caused by a nutrient deficient during lactation [12]. To compensate that deficit, smaller piglets might use tail biting to chase other piglets from the feeder or another resource with restricted access [9].

For a deeper insight into the relationship between suckling and rearing behavior, we performed a principal component analysis including each agonistic suckling behavior and manipulative rearing behavior separately. The highest contribution to principal components 1 and 2 related to all pigs were made by the agonistic suckling behaviors, independently of the success or intensity of the described interaction. However, the contributions of manipulation behaviors are clearly smaller than those of suckling behaviors to both principal components. This result suggests that the pigs might be primarily similar in their suckling behavior. This may be explained by the differences of the behavioral recording during both suckling and rearing. In the suckling period, the agonistic interactions were only recorded during the short durations of the suckling bouts, but not for the remaining time. Therefore, less variation in behavioral expressions is expected to occur compared to the traits recorded during rearing.

By separation of the pigs into two clusters, principal components of cluster 1 included main contributions of five different suckling behaviors, received tail biting and performed ear biting behavior, whereas principal components of cluster 2 were similar to principal components related to all pigs and thus primarily directed by the agonistic suckling behaviors. Since no significant correlation between those suckling and rearing behaviors was found and, furthermore, the pigs of a cluster did not fit properly in their cluster (silhouette width = 0.27), we cannot derive a reliable connection between the analyzed agonistic suckling behaviors and manipulative rearing behaviors of pigs from this type of analysis.

## 5. Conclusions

In our study, tail lesions were detected with a delay of about ten days, when an increase of tail biting behavior had already occurred. This underlines the importance of behavioral analysis for early detection and prevention of tail biting. The early identification of pigs predisposed for tail biting is hampered by the observed repeated shift in roles between biters and receivers, as also described by Zonderland et al. and Hoy et al. [10,11]. However, the observed moderate correlation between dominance index during suckling and performed tail biting during rearing warrants further research. To analyze further facets of tail biting behavior for the identification of pigs predisposed to tail biting, more aspects of pig personality as shown in early life should be considered.

## Figures and Tables

**Table 1:** Number of pigs, mean weaning and rearing weight ± SD, number of dams and sex ratio per rearing pen.

Repetition	Pen	Weaned Pigs (N)	Average Weaning Weight (kg)	Dams (N)	Sex Ratio Male:Female	Average Weight at the End of Rearing (kg)
1	1	9	6.12 ± 0.21	4	4:5	19.79 ± 2.43
	2	10	6.74 ± 0.26	5	6:4	22.03 ± 2.62
	3	10	7.30 ± 0.22	5	5:5	20.97 ± 2.50
	4	10	7.80 ± 0.17	5	5:5	23.06 ± 2.34
	5	10	8.53 ± 0.23	5	5:5	25.34 ± 2.63
	6	10	9.38 ± 0.31	4	5:5	25.72 ± 2.55
2	1	9	6.60 ± 0.23	5	4:5	21.20 ± 3.13
	2	10	7.33 ± 0.28	5	5:5	21.23 ± 3.46
	3	10	7.96 ± 0.15	5	5:5	22.53 ± 2.86
	4	10	8.40 ± 0.20	5	5:5	22.51 ± 2.01
	5	10	9.08 ± 0.27	5	5:5	23.66 ± 3.00
	6	10	9.58 ± 0.24	4	5:5	25.10 ± 1.84

**Table 2:** Scoring scheme of the general health condition (modified after [43]).

Parameter	Score 0	Score 1	Score 2
Lameness	No lameness	Lameness	-
Injury of the body sides	No injury	One side injured	Both sides injured
Injury of the carpal joints	No injury	One side injured	Both sides injured
Injury of the ears	No injury	One side injured	Both sides injured

**Table 3:** Scoring scheme for possible tail lesions (German pig scoring system (Deutscher Schweine Bonitur Schlüssel) of FLI [44], modified).

Parameter	Score 0	Score 1	Score 2
Tail length	Original length	Partial loss	Total loss (stump max. 1 cm)
Hair coat	Covered	Not covered	-
Cleanliness	Clean	Dirty	-
Skin perforation	No visible perforation	Superficial punctual perforation	Deeper skin perforation
Blood	No blood	Fresh blood	-
Necrosis	No necrosis	Necrosis, tissue change	-

**Table 4:** Ethogram of the behavioral traits of the pigs during rearing.

Behavior	Description	Observation Method
Animal directed behavior		
Tail biting	Visible tail biting: biting on the tail of a pig (“victim”) by another pig (“performer”); visible tail-in-mouth behavior: light chewing on the tail of a victim by a performer; new action counted after an interruption of at least 3 s with no contact between the snout of the performer and victim’s tail	Continuously, individual performer and victim, reaction of victim
→ Reaction of the victim:		
Tolerate	No visible reaction as a consequence of tail biting	
Avoid	Pulling in the tail, move away (slides forward while lying) or walking away calmly	
Jump up	Sudden rising of the animal from a lying or resting position; sudden leap forward when the animal was already standing	
Blood	Visible fresh blood on the victim’s tail immediately after a bite; recorded only on first appearance, not on follow-up bites on the same pig when fresh blood was already visible	
Ear biting	Visible ear biting or chewing on the ear of a pig (“victim”) by another pig (“performer”); new action counted after an interruption of at least 3 s with no contact between the snout of the performer and victim’s ear	Continuously, individual performer and victim
Belly nosing	Rhythmically raising and lowering the snout of a pig (“performer”) with pressure in the area of the abdominal cavity of another pig while it was lying; at least 2 s of continuous movement	Scan sampling, performer
Object orientated behavior		
Cotton rope and metal chain with plastic piece (“rope”)	Visible “take in the mouth”, chewing, holding or pulling on one of the components; targeted searching, sniffing or moving one of the components with the snout; visible movement of the components	Scan sampling, performer
Piglet bowl with pellet mix (“pellets”)	Lowered head in or over the piglet bowl; visible manipulation of the bowl, rooting under the outer edge of the bowl, chewing of bowl components or pellets	Scan sampling, performer
Jute sack (“sack”)	Visible “take in the mouth”, chewing, holding or pulling on the jute sack; targeted searching, sniffing or moving the sack with the snout; visible movement of the sack	Scan sampling, performer

**Table 5:** Scored parameters with proportion (%) of pigs for each score.

Parameter	Repetition 1						Repetition 2					
	Day of Life						Day of Life					
	35	42	49	56	63	70	35	42	49	56	63	70
General health condition												
Lameness	2% ^a^	0%^a^	0% ^a^	0% ^a^	0% ^a^	0% ^a^	0% ^a^	0% ^a^	0% ^a^	0% ^a^	0% ^a^	0% ^a^
Body side(s) injured	0% ^a^	0% ^a^	0% ^a^	24% ^b,c^	56% ^d,e^	71% ^d^	37% ^c,e^	0% ^a^	5% ^a^	15% ^b^	38% ^c,e^	59% ^d,e^
Carpal joint(s) injured	61% ^a^	34% ^a^	51% ^a,b^	39% ^a,b^	0% ^c^	5% ^c,d^	49% ^a,b^	31% ^b^	37% ^a,b^	61% ^a^	2% ^c,d^	10% ^d^
Ear(s) injured	15% ^a,b^	5% ^b,c^	12% ^a,b^	2% ^c^	19% ^a^	22% ^a^	80% ^d^	20% ^a^	8% ^a,b,c^	20% ^a^	3% ^b,c^	17% ^a^
Tail lesions												
Fresh blood	2% ^a^	0% ^a^	0% ^a^	8% ^b,c^	22% ^c,d^	36% ^d^	0%^a^	0%^a^	0% ^a^	0%^a^	7% ^b^	15% ^b,c^
Not covered with hair	3% ^a^	0% ^a^	5% ^a^	22% ^b,c^	41% ^c^	51% ^c^	0% ^a^	0% ^a^	0% ^a^	0% ^a^	10% ^b^	2% ^a^
Visible and deeper skin perforation	5% ^a,e^	3% ^a^	10% ^a,b^	17% ^b,c,e^	47% ^d^	64% ^d^	5% ^a^	12% ^a,b^	0% ^a^	15% ^b,c,e^	25% ^c^	47% ^d^
Partial loss	0% ^a^	0% ^a^	0% ^a^	12% ^b^	24% ^b,c^	29% ^c^	2% ^a^	0% ^a^	0% ^a^	0% ^a^	5% ^a^	15% ^b,c^
Necrosis, tissue change	2% ^a,b^	0% ^a^	2% ^a,b^	7% ^b,c^	19% ^c^	14% ^c^	0% ^a^	0% ^a^	0% ^a^	0% ^a^	15% ^c^	2% ^a,b^
Covered with dirt	10% ^a^	25% ^b,c^	27% ^b,c^	37% ^b,d^	44% ^b^	44% ^b^	31% ^b,c,d^	25% ^b,c^	41% ^b^	19% ^a,c,d^	29% ^b,c^	14% ^a,c^

^a,b,c,d,e^ terms with different letters within one row differ significantly by repetition (*p* < 0.05); tested with absolute frequencies by chi-squared test.

**Table 6:** Average manipulation behavior during rearing, frequency ^1^ or percentage ^2^ per pig and hour.

Parameter	Performer Tail Biting (n/h)	Victim Tail Biting (n/h)	Performer Ear Biting (n/h)	Victim Ear Biting (n/h)	Belly Nosing (%)	Rope (%)	Pellets (%)	Sack (%)
Mean ± SD	0.27 ± 0.48	0.27 ± 0.22	0.13 ± 0.11	0.13 ± 0.11	0.30 ± 0.31	3.75 ± 1.38	3.41 ± 1.45	0.20 ± 0.51
Analysis of variance (*p* value)								
Repetition	0.083	<0.001	0.026	0.020	0.005	0.122	0.383	<0.001
Sow	0.187	0.626	0.528	0.286	<0.001	0.002	0.050	<0.001
Sex	0.381	0.928	0.575	0.056	0.278	0.034	0.472	0.658
Pen	0.953	0.008	0.102	0.125	0.002	0.006	0.755	<0.001

^1^ Continuous recording of individual frequency; ^2^ Individual percentage of sampling points.

**Table 7:** Means ± SD and range of Dominance Index and Social Tension Index ^1^ per rearing pen and repetition.

		Dominance Index			Social Tension Index		
Repetition	Pen	Mean ± SD	min	max	Mean ± SD	min	max
1	1	0.08 ± 0.36	−0.58	0.49	−3.33 ± 13.24	−25	22
1	2	0.05 ± 0.24	−0.31	0.45	−6.10 ± 10.56	−27	5
1	3	−0.03 ± 0.33	−0.60	0.35	−2.20 ± 9.60	−15	13
1	4	0.09 ± 0.29	−0.56	0.48	−3.60 ± 9.85	−19	11
1	5	0.20 ± 0.33	−0.12	0.86	1.50 ± 6.98	−8	18
1	6	0.22 ± 0.21	−0.11	0.58	0.90 ± 8.23	−9	18
2	1	−0.08 ± 0.38	−0.51	0.64	−0.11 ± 7.75	−12	11
2	2	−0.07 ± 0.32	−0.67	0.37	−4.10 ± 6.89	−18	3
2	3	0.01 ± 0.18	−0.33	0.29	3.10 ± 6.76	−8	13
2	4	0.06 ± 0.31	−0.47	0.55	−1.70 ± 8.30	−12	14
2	5	0.05 ± 0.35	−0.60	0.67	0.80 ± 7.35	−6	18
2	6	0.03 ± 0.30	−0.39	0.63	−4.60 ± 8.63	−17	8

^1^ Based on individual agonistic traits during the suckling period; for details see Warns [42].

**Table 8:** Spearman’s rho (r_s_) coefficients between Dominance Index and Social Tension Index during suckling, frequencies and percentages of manipulative behavioral traits during rearing and general health condition and tail lesions at the end of the rearing period.

Trait	Rearing Weight	DI ^1^	STI ^2^	Tail Biting Performer	Tail Biting Receiver	Ear Biting Performer	Ear Biting Receiver	Belly Nosing	Rope	Pellets	Sack
Weaning weight	0.517 ***	0.113	0.089	−0.199 *	−0.098	−0.130	−0.089	0.049	−0.132	0.001	−0.030
Rearing weight		0.198 *	−0.020	−0.119	−0.030	0.017	−0.065	0.003	−0.060	−0.072	0.011
DI ^1^			−0.466 ***	−0.256 **	0.073	0.165	0.068	0.000	0.054	0.046	−0.012
STI ^2^				0.148	−0.075	−0.069	−0.042	−0.067	−0.007	0.100	0.070
Tail biting performer					−0.106	0.108	0.037	−0.010	0.037	−0.168	0.059
Tail biting receiver						0.043	−0.071	−0.027	0.036	0.042	0.276 **
Ear biting performer							0.123	0.091	−0.023	0.054	−0.146
Ear biting receiver								0.117	0.123	−0.029	−0.099
Belly nosing									0.086	0.056	−0.059
Rope										0.053	0.244 **
Pellets											−0.029
Blood	0.077	0.048	0.069	−0.135	0.379 ***	−0.034	0.042	0.102	0.024	−0.026	0.062
Hair coat	0.110	0.134	0.041	−0.088	0.265 **	0.033	0.156	0.243 **	0.019	0.043	0.435 ***
Skin lesion	0.042	0.124	0.034	−0.073	0.348 ***	0.141	0.064	0.050	−0.162	0.068	0.051
Partial loss	−0.074	−0.108	0.013	−0.015	0.313 ***	−0.162	−0.177	−0.087	−0.010	−0.097	0.555 ***
Necrosis	0.126	0.201 *	0.048	0.071	−0.051	0.008	0.134	0.104	−0.201 *	−0.098	−0.036
Dirtiness	0.075	0.109	0.043	0.083	0.017	−0.012	0.159	−0.052	−0.084	−0.087	0.062
Injured body side(s)	0.306 ***	0.080	−0.076	0.012	0.140	−0.029	0.062	0.203 *	0.121	−0.124	0.156
Injured carpal joint(s)	−0.113	−0.043	0.168	−0.011	−0.078	0.095	−0.020	−0.045	−0.085	0.129	−0.133
Injured ear(s)	0.130	0.088	−0.031	−0.147	0.147	−0.091	−0.060	−0.001	−0.126	−0.139	0.154

* *p* < 0.05, ** *p* < 0.01, *** *p* < 0.001, ^1^ DI = Dominance Index, ^2^ STI = Social Tension Index.

**Table 9:** Outcomes of the Principal Component Analysis of suckling and rearing behaviors with Eigen value >1.

Behavior	PC 1	PC 2	PC 3	PC 4	PC 5
Eigen value	3.117	1.774	1.447	1.326	1.129
Variance explained (%)	19.484	11.088	9.041	8.286	7.054
Variance explained (%, cumulative)	19.484	30.572	39.613	47.899	54.953
Suckling behavior ^1^					
Push—success	**0.638**	−0.365	−0.267	−0.042	0.194
Push—no success	**0.654**	−0.509	0.042	0.020	0.006
Bite—success	**0.591**	0.494	0.157	−0.147	−0.266
Bite—no success	**0.565**	0.510	0.067	−0.072	−0.282
Push—defeat	**0.716**	−0.378	−0.076	−0.033	−0.010
Bite—defeat	**0.431**	0.348	0.056	−0.008	0.380
Push—defend	**0.733**	−0.149	−0.066	0.017	0.062
Bite—defend	**0.528**	0.507	−0.083	0.228	0.043
Rearing behavior					
Belly nosing	0.168	−0.185	**0.586**	−0.077	0.056
Rope	0.065	−0.195	0.493	**0.586**	0.143
Pellets	−0.175	−0.158	0.000	−0.161	**0.352**
Sack	−0.043	0.039	−0.187	**0.750**	0.106
Tail biting performer	−0.061	0.059	**0.394**	0.377	−0.364
Tail biting receiver	0.035	0.366	−0.264	0.258	**0.497**
Ear biting performer	−0.135	0.313	0.277	−0.291	**0.423**
Ear biting receiver	0.106	−0.008	**0.632**	−0.110	0.272

PC = Principal Component factor; highest loadings per behavior are in **bold type**; ^1^ For detailed explanation of traits see Warns [42].

## Data Availability

Not applicable.
